# Conscious and nonconscious thought: Insights from the neuroscience of decision-making

**DOI:** 10.1073/pnas.2601239123

**Published:** 2026-05-26

**Authors:** Michael N. Shadlen

**Affiliations:** ^a^Department of Neuroscience, Columbia University, New York, NY 10027; ^b^HHMI, Chevy Chase, MD 20815; ^c^Zuckerman Mind Brain Behavior Institute, Columbia University, New York, NY 10027

**Keywords:** intention, reporting-affordance, theory-of-mind, weak phenomenology

## Abstract

Conscious thought is often treated as a special class of neural processing, distinct from the nonconscious computations that guide most behavior. Here I offer a different perspective, grounded in the neuroscience of decision-making. I argue that conscious thought arises not from a unique mechanism but from a distinctive use of neural representations already engaged in nonconscious thought. Nonconscious thoughts are structured as interrogations that yield provisional intentions—decision-like commitments that guide action or inquiry without entering awareness. Using examples from perceptual decision-making and neurophysiology, I suggest that such thoughts depend on persistent neural representations that encode not only potential actions but the questions that give them meaning. These knowledge states may also preserve source-sensitive structure that supports a minimal experiential organization even when they remain nonconscious. Conscious thought, on this account, emerges when such a state is reformatted for potential report to another mind, or to oneself, recruiting theory of mind and narrative structure and placing its content in a space presumed to be shared. This proposal identifies a tractable bridge from nonconscious decision mechanisms to phenomenal consciousness, thereby placing part of the hard problem within empirical reach.

## Significance

This Perspective proposes an operational neurobiological account of thought and identifies a specific transformation by which some thoughts become conscious. Drawing on the neuroscience of decision-making, it argues that thoughts are neural representations of provisional intentions that confer meaning on sensory information and often guide behavior without awareness. Some nonconscious knowledge states may already preserve enough source-sensitive structure to support a minimal experiential character. Conscious thought arises when such states are transformed into representations suitable for shared reference and potential report—to another mind, or to oneself—recruiting theory of mind and narrative structure. This framework offers a tractable bridge from mechanistic neurophysiology to phenomenal consciousness and places part of the hard problem within empirical reach.

A mother sleeps through the din of city traffic but awakens to the faint cry of her baby. The sound is not especially loud, but it is meaningful. Her brain, while still “asleep,” had been monitoring the environment—evaluating sounds, suppressing irrelevant ones, and remaining selectively attuned to those with potential significance. The decision to wake—to re-engage the world—reflects not just arousal, but a transition from nonconscious to conscious processing.^*^ That shift invites us to distinguish two forms of consciousness. One concerns vigilance, sleep–wake transitions, and responsiveness to stimulation—what I will call M−consciousness after the medical approach to consciousness, practiced by neurologists and anesthesiologists.

The other involves awareness, meaning, deliberative engagement and subjective qualities—what I will call P−consciousness, after the philosophers and psychologists who have tried to characterize it. This essay concerns the neural mechanisms responsible for P−consciousness. I will argue that those mechanisms operate on nonconscious thought—structured, goal-directed processes that often remain outside awareness—and that P−consciousness arises when such processes are engaged in a particular way.

## Roadmap.

I begin by proposing an operational account of what constitutes a “thought” in neurobiological terms, seen through the lens of a decision researcher.^†^ I illustrate this with a simple case of nonconscious “thereness,” characterized as a thought structured as an interrogation and formatted as a provisional intention to act or inquire. I then generalize this to nonconscious knowledge states supported by persistent activity in the association cortex, discussing their source-sensitive structure and the possibility of “weak phenomenology.” Next, I argue that conscious thought arises when such a state is taken up as a potential report to a mind presumed to be like one’s own. An *SI Appendix* provides a fuller comparison with other theories of consciousness.

Here, I approach P−consciousness through the lens of decision-making—not in the deliberative or economic sense, but in the neural sense of committing to an action, plan, or proposition based on neural representations of relevant evidence. I will explain the relationship between a state of commitment and thought. Thought itself need not be conscious to guide behavior. Indeed, most thought is nonconscious,^‡^ but some thoughts become conscious. My aim is to understand what neural mechanisms govern that transition.

Let me begin with a thought-experiment about a simple nonconscious thought. Picture yourself in a dimly lit room when a small red light—perhaps the LED of a smoke detector—flickers once and disappears. Because you are reading these words, you are likely imagining the light as you would see it, consciously. Yet that is not the sort of thought I have in mind, not yet. Instead, suppose you failed to notice the flash because your attention was elsewhere—say, reciting the alphabet backward and trying to keep your place. The light appeared just as you reached W, but it escaped awareness. Not noticing is not the same as not seeing: Your eyes were open, photons struck the retina, and neurons in the visual cortex responded. These are the first steps of vision, necessary^§^ but not sufficient for experience. In that moment, you may have seen the light nonconsciously.

To see nonconsciously is to incorporate information about the flash—its location, timing, and other features—into a mental process or disposition to act, without awareness. When the light flashes again, the brain may register it as familiar, a repetition of the earlier disturbance. You might even shift your gaze toward it without realizing you have done so, and still without conscious awareness of the flash itself. Our daily lives are filled with such unnoticed exchanges between perception and action: easing pressure on the brakes at a red light while absorbed in thought, checking for traffic at an intersection without remembering having done so.

These quiet adjustments are decisions without awareness—momentary thoughts that never reach the level of experience. Indeed, the brain can integrate complex contextual information and maintain representations of environmental regularities entirely outside of awareness (3, 4).

To understand what gives rise to such nonconscious thoughts, we can follow the trail of neural activity that the flash initiates. The first stirrings in the cerebral cortex occur in primary and secondary visual cortex, whose neurons respond transiently to the appearance of the light at a specific location in the visual field—its polar angle and distance from the current direction of gaze,{θ,r}. When the flash occurs, populations of visual sensory neurons with receptive fields overlapping that location produce a strong, brief increase in firing rate, but this response alone does not constitute a nonconscious thought.

**Fig. 1.**
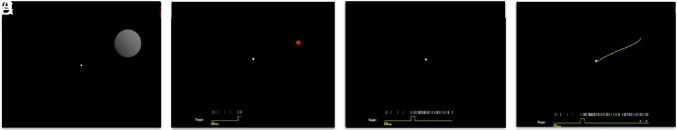
Spiking activity of an LIP neuron during a delayed-saccade task. (*A*–*D*) Key frames from a movie rendering action potentials as audible spikes. The gray region in (*A*) denotes the neuron’s response field (RF). A brief flash in the RF (*B*) elicits persistent spiking (vertical lines) that continues through the memory delay (*C*). (*D*) Final gaze shifts to the remembered location. The persistent activity shown here occurs when an orienting decision is engaged; the LED example extrapolates this mechanism conceptually and does not imply that such persistence accompanies all nonconscious visual processing. See neural thought for the full audio-visual demonstration.

The sensory signals are relayed to association areas in posterior parietal and frontal cortex, including the lateral intraparietal area (LIP) and the frontal eye field (FEF)—key nodes in a broader network that organizes orienting behaviors. In these regions, neurons respond to the flash primarily when it occurs in the context of a potential action. If the disturbance engages an evaluation—Might I inspect that location?—and if the answer is affirmative, a subset of these neurons produces activity that outlasts the sensory burst. This persistence is not an inevitable consequence of the flash, but the physical manifestation of a provisional commitment—a form of decision-related activity observed in the association cortex (5). It is a state of readiness that can continue for seconds. This activity is the product of an interrogation: the brain’s way of holding that commitment insulated from the immediate sensory flux. What begins as a fleeting response to a flash is thus transformed into a durable knowledge state—a “Might I?” that remains valid long after the sensory transient has subsided (Fig. 1).^¶^

This interrogative structure—a question followed by a provisional intention^#^—is a general strategy of the association cortex, not a specialization of LIP. Similar patterns of decision-related activity are observed across frontal and parietal networks, providing a common format for organizing a vast repertoire of actions: to reach, grasp, or withhold. In each case, the underlying logic is the same: The brain selectively evaluates evidence to reach a state of commitment. This preservation of provisional intentions allows the organism to move beyond reflexive responding to a map of what the environment affords.

The same neural phenomenon can be understood from a different intellectual tradition—as an implementation of what J. J. Gibson called affordances: Objects and events, he observed, invite certain kinds of interaction: a handle affords grasping; a stair affords climbing; a flash affords orienting. The concept is often misunderstood as implying that affordances are read directly from the sensory array without computation. Gibson’s deeper point was that what we perceive are the outcomes of computation—namely, those aspects of the environment that matter for action. In neurobiological terms, a percept is the provisional commitment to a course of action that an event or object affords. The association cortex,^‖^ rather than passively depicting the world, interrogates it for opportunities to act. Knowledge, so construed, is a state of readiness—a capacity for utilization. Most of what we know at any moment is of this nonconscious kind. We are not aware of it precisely because it does not demand awareness; it is the background intelligence that allows us to move through the world efficiently. Its neural correlate is the same persistent activity that confers temporal depth, or freedom from immediacy—what Merleau-Ponty called “the temporal thickness of the present” (7).^**^

This framing also clarifies why knowledge and decision-making are so closely allied. To acquire knowledge is to reach a provisional commitment among possible behaviors—to treat one option as, for now, the answer to an implicit question. The brain’s associative circuits continuously interrogate the sensory world: Might I look there? Might I reach for that? The form of each question is determined by the circuit’s connectivity—what sensory sources it can consult and what motor systems it can influence. An affirmative answer yields meaning. Even the simple acknowledgment of thereness, before any recognition of what it is, arises from an affirmative answer to the question, “Might I look there?”

Clinical neurology offers a natural experiment that highlights this idea. Consider two fictional avatars representing distinct lesions: Joy, whose damage is confined to the right visual cortex, and Bob, whose injury lies a few centimeters anterior, in the right posterior parietal cortex. Both have trouble seeing objects on the left, but in strikingly different ways. Joy’s lesion lies in the right visual cortex, producing blindness of the left side of visual space, relative to the current direction of gaze. On clinical examination it is revealed that central vision is spared—a common pattern when the cause is an ischemic stroke, though similar sparing may follow trauma or tumor. Joy’s chief complaint is that she is “having trouble seeing,” that objects seem indistinct or dim, “as though I were looking through a shade.” She soon realizes that the deficit is confined to one side of her visual world. Rehabilitation exploits this awareness. She is taught to look slightly to the left of objects of interest, using cues from what remains visible and from the expected layout of her surroundings.

The strategy works because Joy’s parietal cortex still poses the relevant question—Might I look there?—even though the usual visual pathway provides no answer.^††^

Bob’s lesion lies a few centimeters anterior, in the right posterior parietal cortex. Unlike Joy, his visual cortex is intact, but the apparatus that poses the question—Might I look there?—is impaired. Bob does not complain of blindness, and to some extent he is correct: he can see. Yet he ignores what appears on the left, not just relative to the direction of gaze but to other objects in the visual field and imagined or remembered scenes. When asked to name the shops on a familiar street, he lists only those on the right side. If asked to imagine himself standing at the opposite end of the street, he now names the previously omitted shops—those that have moved, in imagination, to his right (8). His deficit is not a loss of sensation but a loss of interrogation. The question of looking is no longer posed, so the left side of the world—and of thought—ceases to exist for him.

These contrasting cases expose the architecture of knowing. In Joy, the apparatus that asks the question is intact but receives a degraded reply; she experiences darkness—the mark of an unanswered interrogation. Indeed, for reasons I will return to later, aspects of her deficit operate outside awareness while others reach it. In Bob, the apparatus of interrogation itself is gone; without the question, there is no knowledge of absence.^‡‡^

A thought—even a nonconscious one—requires both a question and the provisional intention that represents the answer. When either is missing, knowledge fails to arise. Joy and Bob illustrate complementary aspects of this principle. Joy’s brain continues to ask and answer the orienting question—Might I look there?—and her experience, though impoverished, retains the structure of a nonconscious thought process that could become conscious through a mechanism I will describe later. Bob’s brain no longer poses the question at all, and for him the left side of the world ceases to exist. In both cases, thought reveals itself as an interrogation whose content is shaped by its intention and by the affordance that provoked it. The brain’s provisional intentions—its silent answers to such questions—constitute the raw material of knowing. As the philosopher Hubert Dreyfus once observed, following Gibson and Merleau-Ponty (9),

The agent does not merely receive input passively and then process it. Rather, the agent is already set to respond to the solicitations of things. The agent sees things from some perspective and sees them as affording certain actions. What the affordances are depends on past experience with that sort of thing in that sort of situation.

Dreyfus extends the scope of affordances to possibilities encountered in a world unanticipated by evolution, while retaining the interrogative structure of thought—the sense in which the world solicits action or response. Through learning and abstraction, we acquire new sensitivities to affordances: not merely to what can be grasped, eaten, or fled from, but to what can be used or meant. A recording device affords the possibility of remembering a bird’s song; a notebook affords the possibility of preserving a thought; a computer cursor affords the possibility of moving something that does not physically exist.

These examples illustrate that the brain must learn new ways of asking its own “Might I...?” questions. Although evolution could not have endowed us with dedicated circuits for inspecting recordings or moving cursors, the same neural machinery that supports perceptual inquiry can be adapted to such novel interrogations. Each learned affordance recruits a provisional intention, linking what provokes the question to what the organism is poised to accomplish. Whether that intention is sustained depends on a decision process—an internal commitment that the opportunity is worth pursuing. If the answer to “Might I...?” is no, the transient activation dissolves; if yes, it stabilizes as a knowledge state.

The content of such a state includes the provoking affordance, the provisional intention it elicits, and any associated perceptual or contextual features that make the pairing meaningful. In our experiments on perceptual decision-making, for example, a dynamic display of random dots affords the possibility of obtaining a reward by looking to one of two choice targets. The content of this knowledge state includes the provoking dynamic stimulus in the context of the experiment, the positions of the choice targets, {θ,r}, and the evolving confidence that the chosen action will be rewarded.

That same structure—the coupling of a provoking affordance, a provisional intention, and its associated features—applies to another special affordance that is likely unique to our species: the possibility of reporting to another mind similar to mine. For Homo *sapiens*, every event, every experience, every nonconscious thought and knowledge state affords the possibility of reporting it to another being—or myself with a mind that I presume to be similar to mine. The possibility of telling, asking, or showing thus becomes itself a kind of provoking affordance, one that recruits the same circuitry of intention, evaluation, and commitment that governs thought and action more generally.

Consider the provisional intention to report—an affirmative answer to the internal inquiry: ‘Might I report this nonconscious thought to another mind like mine? I don’t have to carry out the gesture or verbal act, just as I don’t have to shift my gaze to the flash to know its location. Nor does it matter if the intended recipient actually has theory of mind. What matters is that the reporter presumes it to be so. The provisional intention to report about X gives rise to a knowledge state structured like the nonconscious thoughts, but my claim is that this provisional intention to report makes this nonconscious thought conscious.

Reporting is a natural extension of the decision architecture. The logic of the proposal is already accepted implicitly in the field of nonconscious cognition, where practitioners have developed sophisticated methods (e.g., ref. 10) to probe mental operations that transpire without conscious awareness, which they validate by ensuring that participants cannot report key components of the task.

The notion that reporting [to another mind] implies consciousness is the contrapositive of the assumption that the inability to report implies absence of conscious awareness.

More importantly, the intention to report [to a mind like mine] transforms the knowledge state by augmenting the content of the nonconscious thought to a communication about it. In prelinguistic ancestors this might be a gesture whose interpretation is assumed common to reporter and receiver. With verbal communication it becomes story, warning, directions, origins of objects, and so on.

Reporting is a social act, one that ordinarily presupposes a recipient with a mind like—albeit not exactly like—the reporter’s own. To communicate effectively, the reporter must entertain a model—explicit or implicit—of what the listener can understand, what the listener knows or expects. This stance aligns with what cognitive scientists call theory of mind (ToM), The crucial point is that the reporter’s provisional intention logically presumes it: The act of reporting is framed as if the receiver were capable of understanding and responding in kind. The intention to report can also be to oneself, be it in the form of a provisional report to one’s future self or a silent rehearsal. This internal framing does not depend on a literal listener; rather, it reflects the evolutionary architecture of a social brain. The provisional intention to report—even in isolation—serves as the functional catalyst that crystallizes a transient sensory event into a durable, reportable knowledge state.

Box 1. Theory of mind

The notion of theory of mind invoked here is not limited to explicit reasoning about another person’s beliefs, but to the more general capacity to represent mental states as belonging to an agent with a particular perspective. In the familiar Sally–Anne task (11, 12), for example, a child, Anne, sees that an object is moved to a new location while Sally is absent, and must predict where Sally will search upon her return. Success requires recognizing that Sally’s belief reflects what she has seen, not the current state of the world or Anne’s own knowledge. The critical operation is not tracking an action but maintaining a representation of another agent’s perspective when one’s own information has changed. I suggest that this same representational stance is recruited when a thought is framed as reportable—whether to another person or to oneself—by treating it as content addressed to a mind presumed capable of understanding.

**Fig. 2.**
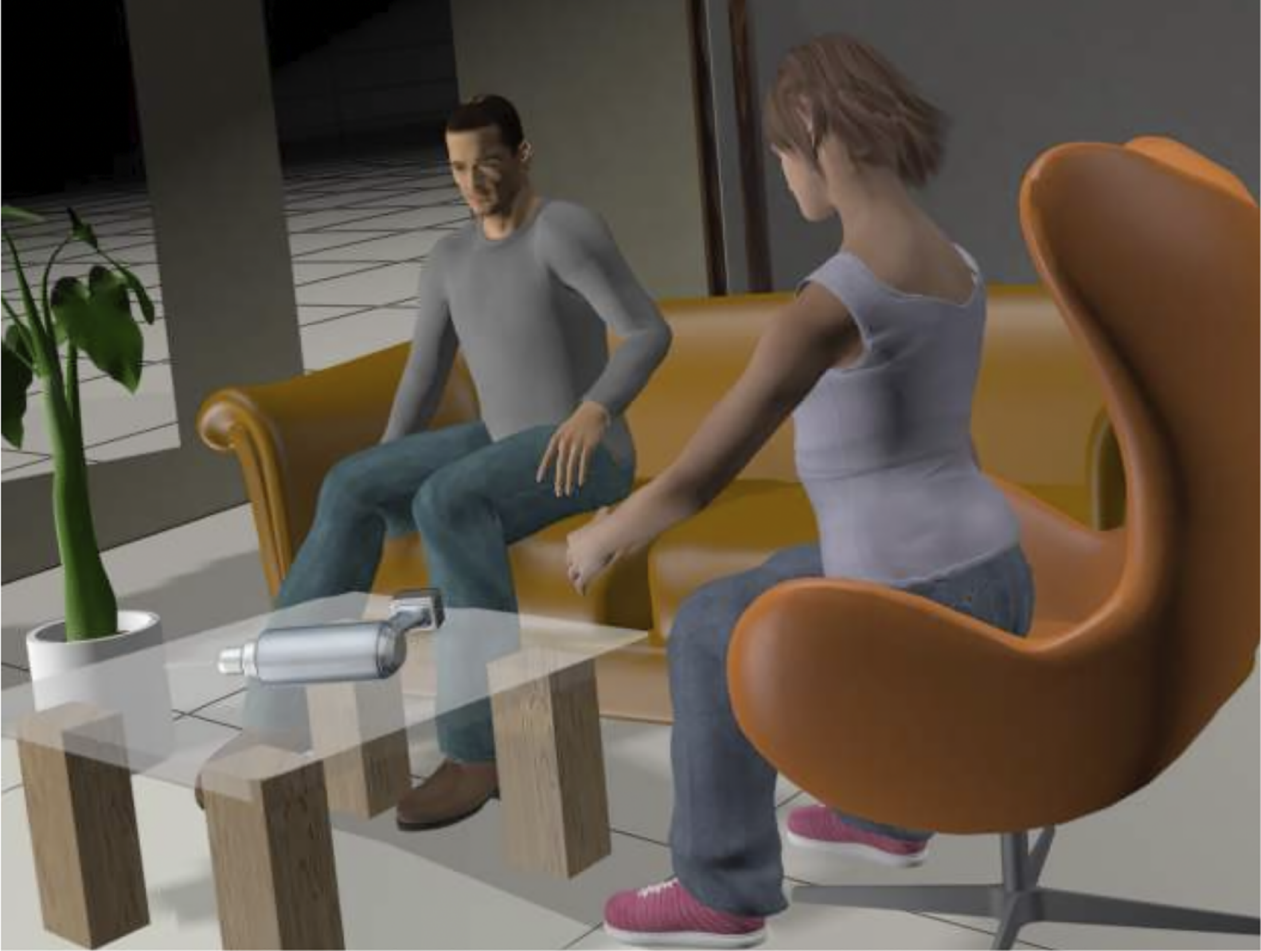
A drill on a table between two minds. The same object supports nonconscious knowledge structured for action and, when framed for report, a knowledge state that sustains shared reference across perspectives.

The intention to report to another mind transforms the content of thought profoundly. Consider the carpenter depicted in Fig. 2. As she examines the power drill on the table, her brain computes its position relative to her eyes and hands, its texture and shape, and the affordances of grasping or avoiding. Because she is a skilled carpenter, her intentions are colored by expectations of the weight of the drill, the gauge of the bit, and the anticipated resistance of the wood. The drill affords the possibility of making a hole in the wall, of scratching or breaking the table, and of causing injury. These possibilities define a field of provisional intentions—answers in waiting to questions such as “Might I avoid dropping, impaling, or electrocuting myself?”

Much of this thought process could unfold nonconsciously, yet it organizes a knowledge state far richer than a mere percept. It encompasses the object’s properties, its potential uses and risks, and the provisional intentions that tie perception to action. It is already a rich knowledge state, but it does not require consciousness. Crucially, none of this yet requires that the drill be represented as something that could be referred to, described, or understood by another mind.

But if the carpenter now entertains the possibility of describing the drill to someone else across the table, her neural representation changes. The drill now occupies not only her own field of possible action but also that of another mind. To support this possibility, the knowledge state must do more than guide action: It must sustain reference. The drill becomes something that can be identified, explained, warned about, or remembered—a thing presumed to remain the same across perspectives and across time.

In this sense, the drill becomes simultaneously private and public—an object situated in a world assumed to be shared. In that instant, she experiences not merely the drill’s affordances, but the shared world it implies: a reality that exists between observers rather than within either alone. This shift in the structure of knowledge marks the transition from a thought concerned with instrumental interaction to one framed for communication—a prelude to consciousness as a decision to report.

Before turning fully to theory of mind and reporting, it is useful to pause over what has already changed within the knowledge state itself. Even prior to report, and even when thought remains nonconscious, the provisional commitments described above are not merely computational. They carry a minimal experiential aspect—what I will refer to as weak phenomenology.

By weak phenomenology I do not mean awareness, reportability, or narrative experience. I mean a sparse what-it-is-like that accompanies certain provisional intentions without making them conscious. When a neural system answers the question “Might I look there?” affirmatively, the resulting knowledge state is not a neutral data structure. It is like other states that would disappear if the eyes were closed, that would change with movement of the body, and that would require approach before contact could be made. This is the weakest sense in which something can be said to be there for the organism.

I earlier referred to this as thereness: the sense that something is present at a particular location in the world prior to identification, recognition, or conscious inspection. Thereness reflects not what an object is, but how it would matter under possible actions. It encodes counterfactual structure—how the information would change if one acted—without yet invoking experience of an object as such. Importantly, such states can guide behavior and learning while remaining outside awareness, and thus belong properly to nonconscious thought.

Weak phenomenology, in this sense, is not yet consciousness. It does not entail that the organism could report what is present, only that the information is available as having come from somewhere—as visual rather than tactile, as distant rather than within reach. It is this minimal experiential aspect that makes it coherent to speak of nonconscious thought rather than mere signal processing. Conscious thought, I will argue, arises when such states are further transformed by a provisional intention to report—to place the content in a space presumed to be shared with another mind.

The dual reference—to world and to other mind—may be the essential signature of conscious thought. It links the mechanisms that represent the world to those that represent agents within it. Neurons that encode the direction of gaze, the intention to act, or the confidence in a decision participate in the first kind of reference; circuits that model the expectations and reactions of another mind participate in the second. When these systems converge, the resulting knowledge state occupies a relational space between self and other. It is not merely about things in the world, nor solely about one’s own possible actions, but about the shared significance of those things. This view resonates with ethical and metaphysical ideas articulated by Martin Buber, who emphasized “the primacy of the between” (13, 14). Buber saw that human cognition evolved in social groups, where meaning and reality arise in relationship rather than isolation.

Adopting this relational stance alters both meaning and motivation. A perception or decision that, moments earlier, served an instrumental goal—grasping, avoiding, or selecting—now acquires an additional dimension: the potential to be understood. The brain that prepares to communicate begins to construct its reality as something that can be jointly known. In this sense, consciousness arises not within the solitary mind but in the between—the space where minds align their representations of the world. It is here that subjective experience acquires its peculiar vividness, as if the world itself were affirming its presence to more than one observer.

The transformation I have described does not depend on overt communication. The intention to report can be directed toward another person, an imagined listener, or oneself, and it need not culminate in speech or gesture. Theory of mind, in concert with narrative, profoundly alters the knowledge state associated with the reporting affordance. It can take the form of a provisional report to oneself—a rehearsal framed for a mind presumed capable of understanding. In her novel, White Teeth, Zadie Smith captures the phenomenology of this transition, describing “the moment in the brain between thought and speech” as a fleeting but charged interval (15, Chp.-2). What matters here is not the literary framing, but the cognitive one: a rehearsal state in which thought is already formatted for understanding and potential sharing. In this way, the reporting stance transforms even simple thoughts into narrative or pedagogical content, placing them in the relational space that spawned human consciousness.

## Implications & Limitations

The transition from nonconscious processing to conscious thought is not a move toward a new kind of “stuff,” but a shift in the use of existing machinery. Specifically, the neurobiology of decision-making—traditionally studied as a bridge between sensation and action—offers a tractable blueprint for consciousness itself. Consider the M−consciousness described at the outset: The mother’s brain evaluates the baby’s cry as a signal of high significance, triggering a global state change—an awakening. We may regard P−consciousness as an analogous state change, albeit one triggered by a different kind of significance.

On this view, the “intention to report” is a formal commitment to a knowledge state within a social-representational space. Just as a motor system stabilizes a plan before execution, the reporting stance stabilizes a representation for communication. This suggests that the circuitry of awakening (M−consciousness) and the circuitry of social reporting (P−consciousness) are not merely overlapping; they are shared motifs of a single, interrogative architecture where the “decision to engage” is the fundamental mechanism of awareness (16).

The central claim of this essay is that many phenomena commonly associated with conscious thought arise from how information is used—specifically, from cognitive operations that treat representations as reportable, shareable, or interpretable by a mind. Decision-making provides a concrete domain in which many supporting operations can be studied mechanistically. The intention to report, whether to another person or to oneself, recruits theory of mind and narrative structure, altering the epistemic status of internal representations.

By treating thoughts as potential reports to a mind presumed capable of understanding, the intention to report does more than make information communicable. It situates cognition within a narrative space in which reasons can be offered, commitments revised, and meanings stabilized over time. This capacity to frame thought for a mind like one’s own may be central to what distinguishes deliberative, reflective thought from other forms of intelligent behavior.

The view that consciousness arises in the relational space between minds—when thoughts are framed as potential reports to another—invites a natural question: To what extent do other animals, or even human infants, share this capacity? The answer likely depends on the degree to which a system can represent another mind’s understanding—that is, its capacity for theory of mind. Evidence from developmental studies suggests that theory of mind is graded rather than all-or-none (17) At one end are organisms that communicate effectively yet rigidly, without evidence of mental representation in either direction. A rodent pup’s distress call, for example, reliably elicits maternal retrieval, but neither party need conceive of the other as having a mind. The exchange is biologically adaptive but cognitively closed.

That said, this stance does not require that nonconscious knowledge states be devoid of phenomenological character altogether. This minimal experiential aspect—weak phenomenology—does not entail awareness, but rather the sparse “what-it-is-like” of a poised intention: the experience not of an object as such, but of there being something to be acted upon.

What makes weak phenomenology scientifically useful is that it already depends on mechanisms that register the provenance of information (source-tagging)—whether a representation arose from vision, touch, or memory—and on expectations about how that information would change under action. Such source-tagging therefore plausibly occurs in animals that lack theory of mind or conscious thought. In this sense, weak phenomenology points to neural operations that are likely within reach of contemporary neuroscience.

### Speculative Note.

The appeal to phenomenology in this essay is not meant to resolve the question of why experience feels like anything at all, but to impose a constraint on any neural account of thought: information that is usable, reüsable, and reportable must preserve the format of how it was acquired (i.e., its source and reüse potential). The question, then, is what kinds of circuit operations could support the reïnstantiation of such source-sensitive knowledge states without rendering them conscious or reportable. To the extent that weak phenomenology depends on registering the source and potential reusability of information, mechanisms involved in learning and credit assignment offer a plausible starting point. This requirement for credit assignment in deep hierarchies necessitates robust feedback projections. Notably, these same pathways provide the substrate for mental imagery—the internal synthesis of experiential content in the absence of bottom–up input (18–20). When these top–down signals are stabilized and source-tagged, they provide the ‘weak phenomenology’ that underlies nonconscious thought.

Long-range feedback projections, which selectively target neurons that were recently active, could in principle provide such tagging without rendering information reportable. Dendritic calcium plateau potentials, triggered by backpropagating action potentials, have been implicated in marking neurons for future plasticity and receptivity (21, 22).

These types of active dendritic currents provide a concrete example of how information might be flagged as having come from a particular source and preserved for reüse—supporting the counterfactual structure characteristic of weak phenomenology. Establishing these cellular mechanisms for weak phenomenology does not instantly bridge the ‘Hard Problem,’ but as argued below, it is a step in that direction.

Establishing these cellular mechanisms for weak phenomenology does not instantly bridge the Hard Problem, but as argued below, it is a necessary step in that direction. If such operations—persistent activity and dendritic tagging—underlie the emergence of a minimal experiential structure, they provide the essential substrate for phenomenal experience. From this perspective, the question of “what-it-is-like” is not a separate ontological mystery, but a structural consequence of reformatting this foundational information for the reporting stance.

### The Reporting Stance and the Hard Problem.

Philosophers often distinguish between explaining the functional and neural mechanisms underlying cognition and explaining why those mechanisms should be accompanied by subjective experience at all. Thomas Nagel famously captured this latter challenge in the phrase “what it is like” (WIL) to be a conscious organism (23). David Chalmers later termed the explanatory gap between neural process and WIL the “Hard Problem” of consciousness (24).

The account developed in this essay does not deny the force of that problem. Nor does it treat WIL as an illusion to be eliminated or a mere byproduct of linguistic practice. Rather, it begins from the observation that certain nonconscious knowledge states already possess a minimal experiential structure—what I have called weak phenomenology. When a neural system answers the question “Might I look there?” affirmatively, the resulting provisional commitment is not a neutral data structure. It carries information about its source, its spatial and temporal relation to the organism, and how it would change under possible actions. This sparse “thereness” does not yet constitute full conscious awareness, but it identifies a form of experiential organization already present in nonconscious cognition.

A useful analogy comes from motor control. Neural activity representing an intention to flex the elbow closely resembles the activity that produces the movement itself; the action occurs when inhibitory gating is released. The intention state is therefore not merely a symbolic representation of movement but a poised form of the movement itself. In an analogous way, certain knowledge states may already possess structured experiential organization even when they are not yet taken up under the reporting stance that characterizes conscious thought.

While this emphasis on the relational character of thought shares ground with accounts that treat awareness as a social or predictive model (25, 26), the present proposal differs by treating experiential structure not as something to be explained away, but as the inherent format of information poised for report (see *SI Appendix*, section B for a fuller comparison).

This perspective suggests that the “Hard Problem” arises not from a gap in physical explanation, but from a failure to account for the structural transformation required for shared reference. We do not need to explain how a neural process “secretes” a private feeling. Instead, we must explain why a knowledge state, when reframed for potential report, necessarily acquires the properties we call qualia. These properties—the “redness” of red or the “thereness” of a thought—are the functional consequences of preparing an internal state to be shared with another mind.

This reframing does not dissolve the metaphysical “remainder” of the Hard Problem; it does not explain why neural processes should be accompanied by any experiential aspect at all. But it narrows the explanatory gap by identifying neural operations—such as persistent activity supporting provisional commitment and source-tagging—that already instantiate a minimal experiential structure. If consciousness is to be explained scientifically, it will likely be by understanding how such structures are stabilized, integrated, and repurposed for shared reference and report.

In this sense, the proposal places part of the Hard Problem within the crosshairs of neuroscience, distinguishing between the existence of experiential structure and the transformation that renders it conscious in the full sense of being reportable, shareable, and narratively integrated. Whether the remaining metaphysical question can be resolved remains open; what can be addressed empirically is how structured nonconscious thought becomes the kind of thought for which there is “something it is like” to think. In this light, the path to a neurobiology of experience follows the logic of the Taniyama–Shimura conjecture. Just as proving that conjecture for the class of semistable elliptic curves provided the essential bridge to Fermat’s Last Theorem, elucidating the mechanisms of weak phenomenology—which are tractable in nonconscious systems and animal models—is the necessary prerequisite for solving the “Hard Problem.” If the neural basis for source-tagging and for encoding the dependence of a commitment on evidence and task contingencies can be established for nonconscious thought, full phenomenal consciousness is likely to follow on its coattails.

## Supplementary Material

Appendix 01 (PDF)

Movie S1. Full audio-visual movie for the example in Figure 1.

## Data Availability

All study data are included in the article and/or supporting information.

## Footnotes

^*^Throughout this article, I use the term “nonconscious” rather than the more common “unconscious” to emphasize that the processes under discussion are not repressed, hidden, or categorically distinct from conscious thought, but differ in how their contents are accessed and used.

^†^A temporally extended internal state of a cognitive system that encodes a structured relation to possible states of the world. Such states are typically poised, in principle, to guide inference, action, or report.

^‡^As Stanislas Dehaene and others have argued, the bulk of cognitive operations—from linguistic parsing to the weighing of evidence—is conducted nonconsciously (1). Conscious access is the exception, not the rule, of neural processing.

^§^Blind-sight is a rare exception; see ref. 2.

^¶^Primates make several saccades per second during natural viewing (6), yet observers are typically unaware of most of these eye movements and of the processes that generate them.

^#^Here, “intention” is used broadly to include provisional commitments that need not culminate in action or decision, but may instead serve to inquire—maintaining a state of readiness to harvest further information from a specific location.

^‖^For Gibson, these are typically subcortical sensory-motor structures. This is at least part of the reason that Gibson used the term, *direct perception*. Cognition is associated with encephalization of the process to cortical association areas—same motif but less immediate.

^**^The temporal persistence Merleau-Ponty emphasized is often discussed in contemporary neuroscience under the rubric of working memory.

^††^In truth, the parietal cortex is not acting in isolation. Even when primary visual cortex (V1) is compromised, alternate routes—from the retina to the superior colliculus and onward through the pulvinar to posterior parietal cortex—can still signal the presence of a stimulus and thus prompt the interrogation. These subcortical pathways are fast, coarse, and nonconscious, yet they can nonetheless instruct association cortex to “ask” the question that visual cortex fails to answer.

^‡‡^What is termed anosognosia—ignorance of one’s disease.
