# Supplementary material for: Conscious and nonconscious thought: Insights from the neuroscience of decision-making
Source: Proc Natl Acad Sci U S A. 2026 May 26;123(22):e2601239123. doi: 10.1073/pnas.2601239123 (PMC13229189; doi:10.1073/pnas.2601239123)
Supplement: Supplementary file 1 — Appendix 01 (PDF) [file pnas.2601239123.sapp.pdf]

Supporting Information for

Conscious and non-conscious thought: insights from the neuroscience of decision making

Michael N. Shadlen

Michael N. Shadlen.
E-mail: shadlen@columbia.edu

This PDF file includes:

Legend for Movie S1
SI References

Other supporting materials for this manuscript include the following:

Movie S1

Appendix. Relation to other theories of consciousness

A. What this account is not. Many contemporary accounts treat consciousness as arising when an existing neural representation is sufficiently amplified—through increased firing rate, synchrony, persistence, attentional gain, or spatial spread. On such views, the distinction between conscious and non-conscious processing is largely quantitative: consciousness reflects a strengthened or globally broadcast version of an underlying representation.

The present account shares with these views the observation that conscious thought is often accompanied by increased stability and widespread cortical engagement. In paradigms such as perceptual masking, the transition to reportable awareness frequently coincides with late "ignition"-like dynamics. Such ignition closely resembles what, in the neuroscience of decision making, is described as commitment: the stabilization of a representation sufficient to guide action or report.

Where the present account differs is in what this stabilization accomplishes. Non-conscious knowledge states—such as provisional intentions supported by persistent activity in association cortex—may already exhibit amplification, persistence, and broad recruitment, yet remain non-conscious. What distinguishes conscious thought is not amplification per se, but a change in how the representation is used: it is reframed under the affordance of report to a mind presumed to be like one's own. Amplification may enable access to this architecture, but it does not by itself define the transition.

This account is not a version of global neuronal workspace (GNW) theory, in which consciousness arises when information is broadcast widely across the cortex. Although the transition to conscious thought described here may coincide with widespread cortical engagement—particularly when reporting is overt or linguistically elaborated—such broadcast is not constitutive of consciousness. What matters is the commitment to a reporting stance, implemented through decision-like mechanisms that stabilize a knowledge state for potential sharing. In this sense, global activation may be a frequent consequence of conscious thought, but not its defining cause. GNW provides a robust account of global access, but without the structural reformatting of the reporting stance, it leaves the Hard Problem as an unexplained 'remainder.' By contrast, the present account suggests that this perceived gap is the result of the brain's internal model of its own reporting affordances.

Although the framework presented here is grounded in the neuroscience of decision making—a domain with deep mathematical structure—it does not identify consciousness with abstract quantities such as entropy, complexity, or free energy. The relevant commitments are evolutionary and mechanistic: how neural systems acquire, stabilize, and repurpose knowledge states for action and communication. The account aims to specify a tractable target for neuroscience, not a unifying physical measure of consciousness.

B. Relation to Attention Schema Theory (AST). The present account bears an important family resemblance to Graziano's Attention Schema Theory (AST) (1, 2). Both views emphasize the social and relational dimensions of consciousness and place theory of mind at the center of awareness. In both frameworks, consciousness is not treated as an intrinsic, irreducible property of neural activity, but as arising from how the brain models or deploys its own internal states in a social context. Each account rejects the idea that qualia must be explained as ontologically primitive ingredients.

The crucial difference lies in the target of the modeling. In AST, the brain constructs a simplified model—a "schema"—of the process of attention, which it uses to explain and predict behavior. Consciousness, on this view, is the attribution of an attentional state. By contrast, the present account focuses on how knowledge states themselves are transformed when they are formatted for potential report.

While AST describes a model of the process of awareness, the reporting stance describes a structural reformatting of the content. On this view, "qualia" are not illusory attributes of a schema; they are the functional properties—such as source-sensitive tags and stabilized commitments—required for a knowledge state to be shared with a mind presumed to be like one's own. We do not merely model an internal state of awareness; we adopt a relational stance toward our own knowledge, transforming "weak phenomenology" into the stable, interpretable objects of conscious thought.

C. Relation to Higher-Order and Report-Based Accounts. Higher-order theories of consciousness propose that a mental state becomes conscious when it is represented by another mental state that attributes that experience to oneself—for example, a thought to the effect that I am seeing X. On these views, consciousness depends not on first-order perceptual representations alone, but on the presence of a distinct meta-representational layer. Empirical motivation for such accounts has often been drawn from dissociations between perceptual performance and subjective confidence or awareness (3, 4).

The present account shares with higher-order theories an emphasis on metacognitive structure and on the role of self-related representation in conscious thought. However, it differs in a key respect. It does not posit a second representation that represents a mental state as such. Instead, the same knowledge state is transformed when it is taken up under a different functional affordance: the possibility of report, either to another mind or to oneself. Consciousness arises not because a mental state is re-represented, but because it is used in a role that presumes interpretation, understanding, and potential sharing.

In many experimental contexts, reportability is treated as an operational criterion for consciousness: a stimulus is classified as consciously perceived if the subject can report it, and as non-conscious if report fails. This convention is useful methodologically but does not itself explain what distinguishes conscious from non-conscious processing. The present proposal treats reportability differently. Rather than serving as a diagnostic test, the possibility of report is part of the functional transformation that gives rise to conscious thought. A knowledge state becomes conscious when it is taken up under the affordance of communicating that knowledge—either to another mind or to oneself.

On this view, metacognitive features such as confidence—already present in many non-conscious knowledge states (5, 6) can be incorporated within the same state rather than supplied by a separate monitoring representation. Consciousness thus reflects a shift in epistemic role rather than the addition of a distinct representational layer.

D. Modeling Perception as Covert Behavior. Markkula's proposal to model perception as covert behavior (7) represents one of the closest antecedents to the present account. Like the view developed here, it emphasizes continuity between perception and action, rejects intrinsic qualia as explananda, and treats reports of experience as phenomena to be explained rather than as direct windows onto intrinsic qualia. In Markkula's framework, the selection of narrative behavior—covert or overt—plays the central explanatory role. By contrast, the account advanced here places provisional intention and decision-like commitment prior to narrative description.

While Markkula identifies the "what" of the behavior (the narrative), the reporting stance identifies the "why" of the experience: the transformation occurs because the commitment is formatted for a mind presumed to be like one's own. This suggests that the "covert behavior" of perception is not merely a rehearsal of words, but a structural realignment of information into a shareable format—a transition from a private estimate of evidence to a public-facing claim. A thought, whether conscious or non-conscious, arises when an implicit question (e.g., Might I look there? Might I act?) is answered affirmatively and stabilized in a knowledge state insulated from immediacy. This shift has two consequences. First, it provides a direct bridge to the neurobiology of decision making, where persistent activity in association cortex has been shown to encode provisional commitments that guide behavior. These decision-related knowledge states already possess structure, counterfactual sensitivity, and—using the terminology introduced here—weak phenomenology. Narrative capacity is therefore neither required for non-conscious thought nor sufficient for consciousness. Second, it clarifies the role of reporting. Whereas Markkula treats the capacity for narrative behavior as the basis for admitting conscious experience, the present account treats the possibility of report as an affordance that can be taken up by an existing knowledge state. When a state is reframed as something that could be shared, explained, or understood by another mind—or by oneself construed as such—it acquires the epistemic role characteristic of conscious thought. Narrative structure is thus better understood as a consequence of this reframing rather than its cause.

From this perspective, Markkula's framework identifies an important regularity—the close association between consciousness and narrative report—while leaving open the mechanistic question of how such reportability arises from non-conscious cognition. By grounding this transition in decision-like commitment and the affordance of reporting, the present account aims to render that transition tractable within the neuroscience of decision making and non-conscious thought.

References

1. MSA Graziano, S Kastner, Human consciousness and its relationship to social neuroscience: A novel hypothesis. *Cogn. Neurosci.* **2**, 98–113 (2011).
2. MS Graziano, A conceptual framework for consciousness. *Proc. Natl. Acad. Sci. United States Am.* **119**, e2116933119 (2022).
3. SM Fleming, ND Daw, Self-evaluation of decision-making: A general bayesian framework for metacognitive computation. *Psychol. Rev.* **124**, 91–114 (2017).
4. H Lau, D Rosenthal, Empirical support for higher-order theories of conscious awareness. *Trends Cogn. Sci.* **15**, 365–373 (2011).
5. A Kepecs, ZF Mainen, A computational framework for the study of confidence in humans and animals. *Philos. Transactions Royal Soc. B* **367**, 1322–1337 (2012).
6. R Kiani, MN Shadlen, Representation of confidence associated with a decision by neurons in the parietal cortex. *Sci. (New York, NY)* **324**, 759 – 764 (2009).
7. G Markkula, Answering questions about consciousness by modeling perception as covert behavior. *Front. Psychol.* **6**, 803 (2015).

Movie S1. Full audio-visual movie for the example in Figure 1.
